# T-Cell Large Granular Lymphocytic Leukemia as a Cause for Severe Neutropenia

**DOI:** 10.7759/cureus.10186

**Published:** 2020-09-01

**Authors:** Jordan M Minish, Nadine Hamed, Robert Seifert, Amar H Kelkar

**Affiliations:** 1 Department of Medicine, University of Florida, Gainesville, USA; 2 Department of Pathology, Immunology and Laboratory Medicine, University of Florida, Gainesville, USA; 3 Division of Hematology and Oncology, University of Florida, Gainesville, USA

**Keywords:** lgl leukemia, neutropenia, cytopenias, oncology, flow cytometry

## Abstract

Large granular lymphocytic (LGL) leukemia is a rare, indolent disease that can cause destruction of neutrophils. We discuss the case of a previously healthy 63-year-old male who presented with severe, recurrent febrile neutropenia, in whom three bone marrow biopsies over 13 months failed to produce a diagnosis. He presented to our facility with persistent fevers and an absolute neutrophil count of 20 cells/mm^3^ (reference range 1,700-7,000 cells/mm^3^). A fourth bone marrow biopsy did not show clonal proliferation, but T-cell LGL leukemia was diagnosed based on the identification of T-cell rearrangements. We propose that LGL leukemia could be an underdiagnosed cause of severe neutropenia in patients with no overt malignancy or immunosuppressive therapy and that population-based database studies of patients with unexplained neutropenia may reveal more cases of this rare disease class.

## Introduction

Large granular lymphocytic (LGL) leukemia is a rare lymphoproliferative disorder with three recognized subsets: T-cell leukemia, chronic natural killer (NK) cell lymphocytosis, and NK cell leukemia [1]. T-cell leukemia is the most common and can have a wide variety of clinical presentations, most often presenting with neutropenia, anemia, thrombocytopenia, and autoimmune diseases, such as rheumatoid arthritis [2]. T-cell LGL leukemia is primarily diagnosed by clinical criteria combined with peripheral blood flow cytometry and T-cell clonality studies, as it can be missed on routine bone marrow biopsies [1,2]. T-cell LGL leukemia is usually indolent, and patients often do not require treatment [2]. When patients do require treatment, first-line treatment is with immune suppression [1,2].

## Case presentation

The patient was a 63-year-old Caucasian male who presented to our hospital for evaluation and management of persistent, severe neutropenia and recurrent infections requiring intravenous antibiotics. He had previously been evaluated at multiple other hospitals, but neither the etiology of his neutropenia nor an effective therapy could be found. He had a past medical history of malignant melanoma that had been excised 29 years prior to being seen at our facility, squamous cell carcinoma of the laryngopharynx that had been treated with surgical resection and radiation therapy 19 years prior to admission, recently diagnosed heart failure with reduced ejection fraction, splenomegaly of unknown etiology, recent deep vein thrombosis (DVT) and pulmonary embolism (PE) managed with warfarin, and hypothyroidism.

Thirteen months prior to admission, the patient was first seen for recurrent fevers and splenomegaly. A bone marrow biopsy was performed with suspicion for a T-cell lymphoproliferative disorder and work-up included T-cell receptor (TCR) gene rearrangement; however, the sample was insufficient and further studies were not performed. Four months prior to admission at our facility, the patient was again found to be profoundly neutropenic. A second bone marrow biopsy was performed at a local hospital, which demonstrated bone marrow cellularity at 50%-60%. Flow cytometry showed T cells comprised 17.99% of leukocytes with no specific immunophenotypic aberrancy. TCR was performed and was found to be negative on this biopsy. The patient was discharged on antibiotics, but fevers recurred after the completion of antibiotics and he returned to the hospital with pneumonia and concurrent bilateral pulmonary emboli. During this hospitalization, he was evaluated by rheumatology and had an extensive work-up which resulted in positive antinuclear antibody (ANA), anti-double-stranded DNA (anti-dsDNA), anti-cardiolipin IgA, IgG, and IgM, and lupus anticoagulant, in addition to splenomegaly (16 cm) and abnormal echocardiography with left ventricular ejection fraction of 30%. Rheumatology initiated mycophenolic acid, hydroxychloroquine, and prednisone for a tentative diagnosis of systemic lupus erythematosus (SLE), and the patient was again discharged on antibiotics.

Fevers briefly resolved but resumed after the completion of the extended antibiotic course. At that time, he presented to a different facility, where immunosuppressive medications were discontinued and prednisone was tapered on the basis that the patient did not meet criteria for SLE. A bone marrow biopsy was repeated for a third time, again showing mildly hypercellular marrow with trilineage hematopoiesis and decreased or absent storage iron. There was no overt evidence of a primary neoplasm. Bone marrow flow cytometry showed increased monocytic cells (36% of total cells), mildly increased NK cells (5.5% of total cells), and elevated T lymphocytes (63% of lymphocyte population; CD2+, CD3, CD5+, CD7+). No TCR studies were performed. His right tonsil was also biopsied to rule out recurrence of prior squamous cell carcinoma, but the biopsy showed no malignancy and instead demonstrated a polymicrobial infection. He was discharged on antibiotics including treatment for a Clostridium difficile infection, but once again fevers resumed shortly after cessation of antibiotics.

**Figure 1:**
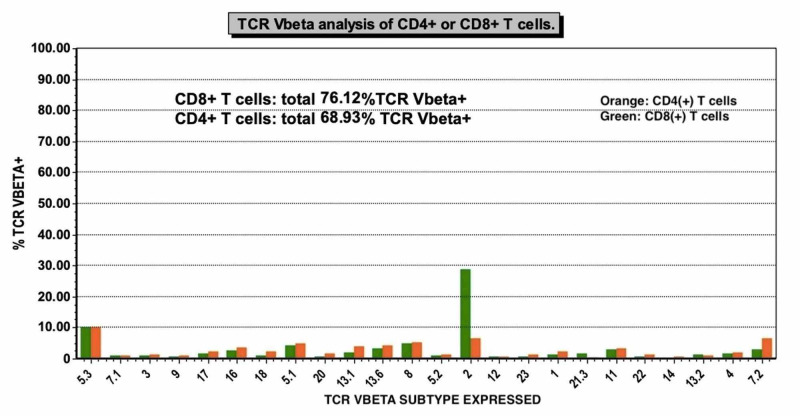
T-cell receptor (TCR) Vbeta analysis on the patient’s fourth bone marrow aspirate. A population of CD3+ and CD8+ T cells was detected that was clonally expanded for Vbeta receptor 2 with all other receptor subtypes being suppressed.

**Figure 2:**
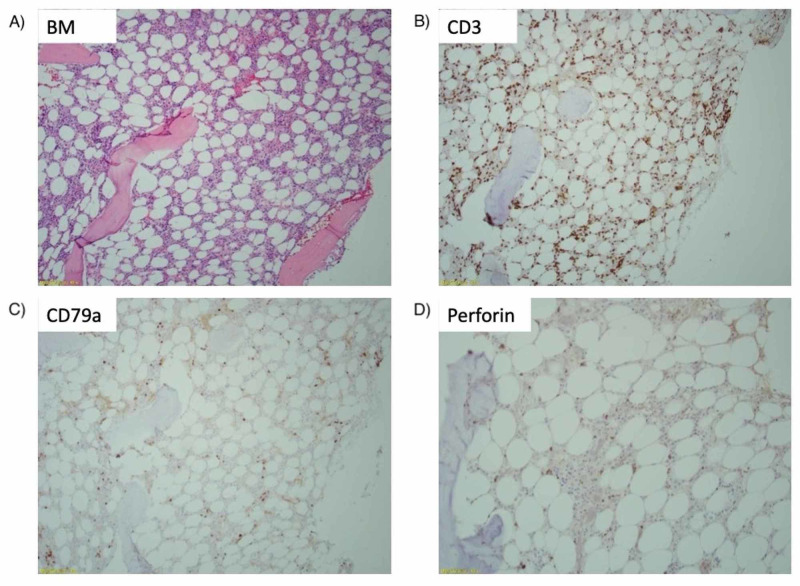
Results of immunohistochemical staining, all figures ×100 total magnification. (A) Bone marrow (BM) H&E stain showing hypocellularity. (B) CD3 immunohistochemistry shows increased T cells in a scattered distribution. (C) CD79a immunohistochemistry demonstrates only rare B and plasma cells. (D) Perforin immunohistochemistry demonstrates an increase in perforin-positive T cells but no infiltrating linear arrays were identified.

The patient then presented to our facility with complaints of sore throat, formation of a black eschar over his right tonsil, and watery diarrhea. He was febrile on admission (T_max_ 39.4˚C) with an absolute neutrophil count (ANC) of 20 cells/mm^3^ (reference range 1,700-7,000 cells/mm^3^) and total white blood cell count of 1,300 cells/mm^3^ (4,000-10,000 cells/mm^3^). Despite completing a full course of antibiotics for C. difficile colitis prior to admission, he remained symptomatic and again tested positive for the stool C. difficile antigen. Oral vancomycin was resumed with a 10-day course planned. During this time, he developed a second tender black eschar on his upper gum line. Oral surgery was consulted and performed a biopsy of the lesion on hospital day 4, which did not show an invasive fungal infection. Hematology and rheumatology were also consulted early in the hospitalization. Hematology reported suspicion of an autoimmune process versus Felty syndrome and recommended additional work-up, including a repeat bone marrow biopsy. Antiphospholipid antibodies and lupus anticoagulant again resulted positive. Due to high clinical suspicion for T-cell LGL leukemia based on the history and review of the peripheral blood smear, the plan was to start cyclosporine following the bone marrow biopsy. The patient’s fourth bone marrow biopsy was performed with submission for flow cytometric analysis and cytogenetic studies. CD3+ T cells were only mildly increased by flow cytometry (24% of cells) and the CD4:CD8 ratio was not significantly altered (1:1). However, TCR Vbeta repertoire analysis was performed by flow cytometry (IOTest Beta Mark Kit, Beckman-Coulter, Brea, CA, USA), which demonstrated a clonally expanded population of CD3+, CD8+ T cells (Figure 1). The bone marrow biopsy also showed an increase in perforin-positive T cells but lacked the classic infiltration by linear arrays (Figure 2). Given these findings and the clinical course, the diagnosis of T-cell LGL leukemia was established. The patient was started on a modified dose of oral cyclosporine at 125 mg and twice weekly granulocyte colony stimulating factor. He was discharged on hospital day 8 in stable condition with an ANC over 690 cells/mm^3^. Ten days later, the patient was no longer neutropenic with an ANC of 1,344 cells/mm^3^. Three months from hospital discharge, ANC was 2,544 cells/mm^3^. Subsequent follow-up was limited due to relocation out of state, but at seven months from hospital discharge, cyclosporine was tapered to 100 mg twice daily by his local physicians with a resulting decrease in the ANC to 500 cells/mm^3^, and it was recommended that he resume prior dosing.

## Discussion

Febrile neutropenia is a well-known cause of morbidity and mortality in patients receiving chemotherapy and other myelosuppressive medications [3]. Risk of severe bacterial and fungal infections, sepsis, and death is significant. Patients with febrile neutropenia due to non-cancerous causes are susceptible to similar infection risks. Unfortunately, the etiology of neutropenia not caused by medication or malignancy can be difficult to diagnose, with a broad differential diagnosis. Therefore, early recognition and evaluation for a diagnosis is critical to the timely initiation of appropriate therapies. In the numerous cases with delayed diagnoses, there is considerable risk for life-threatening infections. In a case of severe neutropenia associated with splenomegaly and positive autoimmune labs, Felty syndrome is a diagnosis that must be considered. Felty syndrome is a disease process that is comprised of seropositive rheumatoid arthritis, splenomegaly, and neutropenia [4]. Our patient did not have a positive rheumatoid factor, which made Felty syndrome a less likely diagnosis. Our patient was ultimately diagnosed with another rare disease process, T-cell LGL leukemia, of which there are many examples of rapid and sustained responses to immunosuppressive therapy, with eradication of neutropenia and infections; however, there is an unmet need for the development of better therapies for LGL leukemia because the disease is currently incurable [5].

LGL leukemia is an extremely rare disease, with an incidence of 0.2 cases per 1,000,000 people. Within LGL leukemia subsets, T-cell LGL is predominant, comprising 85% of patients diagnoses [6]. LGL leukemia has a complicated pathophysiology described by Lamy and colleagues as a disease process at the intersection of clonal lymphoproliferative disease, chronic inflammation, and autoimmunity. It has a significant dysregulation of programmed cell death and an enhanced survival network, centered around the STAT3 pathway. STAT3 dysregulation causes a production of proinflammatory cytokines, leading to autoimmune disease manifestation [1]. There are varying methods of disease treatment, which includes chemotherapy, immunotherapy or hormonal therapy, and combinations of these therapies. Among patients requiring treatment, which is approximately 60%, the overall survival at five years is approximately 62% [7,8]. Among all patients diagnosed with LGL leukemia, the overall survival at 10 years is approximately 70% [1].

Many algorithms for the diagnosis of severe neutropenia recommend bone marrow aspiration and trephine biopsy as an early diagnostic step in patients without obvious predisposing infections or conditions [9]. However, this is a rather invasive procedure that does not always yield a diagnosis. The complete clinical picture should be considered before proceeding to bone marrow biopsy, and less invasive and possibly diagnostic tools, such as peripheral blood flow cytometry, should be discussed. It has been demonstrated that peripheral blood flow cytometry analysis of the TCR beta-chain variable region has a 100% concordance with TCR rearrangement studies of bone marrow aspirates [6]. Additionally, Bareau and colleagues also showed that only 72% of the patients confirmed to have LGL leukemia, who subsequently underwent bone marrow biopsy had bone marrow infiltration with LGLs [10]. This underscores the spectrum of disease findings in this entity. Furthermore, these findings suggest that cases of T-cell LGL leukemia could be missed if the only diagnostic test being used to evaluate severe neutropenia is a single bone marrow biopsy. We suggest that there should be further studies on both peripheral blood and marrow, namely TCR Vbeta analysis and peripheral TCR rearrangement polymerase chain reaction (PCR), and that even with these further studies, patients may require multiple different diagnostic studies to yield a diagnosis.

Unfortunately, there is an unmet need for reliable therapeutic agents for this disease. Currently, the first-line treatment is with single immunosuppressive agents, specifically methotrexate, cyclophosphamide, and cyclosporine. Retrospective analysis of immunosuppressive agents showed a wide variation in overall response rate of 38%-92%, with an equally wide variety in complete remission rate of 5%-50% [8]. Second-line therapy consists of purine analog chemotherapeutic agents, such as fludarabine, cladribine, deoxycoformycin, and bendamustine, which have limited supporting data. Bendamustine was used as a salvage therapy for 20 patients with T-cell neoplasms, with 68% of the patients refractory to first-line therapies. In this trial, bendamustine had an overall response rate of 55%, though only two of these patients had LGL leukemia, with one achieving complete remission and one achieving partial remission [11]. No studies to this point have demonstrated overall survival benefit. At this time, there are many ongoing studies of novel therapies for LGL leukemia, most notably Janus kinase/signal transducer and activator of transcription (JAK/STAT) pathway inhibitors, such as the JAK3-specific inhibitor tofacitinib citrate, and the novel multicytokine inhibitor BNZ-1 [8].

## Conclusions

T-cell LGL leukemia is a rare disease that can range in severity from indolent to severe, with cytopenias resulting in potentially life-threatening infections. It is very difficult to diagnose, often time requiring a multitude of diagnostic studies. As demonstrated in the above case, diagnosis can be difficult without high clinical suspicion, involvement of appropriate specialists, and repeat testing. Three prior bone marrow biopsies did not result in a diagnosis for this patient. The patient’s treatment was thus delayed, and he continued to suffer recurrent infections with lengthy hospital stays and several courses of intravenous antibiotics with associated complications. Diagnosing LGL leukemia is challenging, and often requires multiple studies, be they on peripheral blood or bone marrow, over time in conjunction with a clinical presentation consistent with this disease. TCR Vbeta analysis and TCR rearrangement PCR should always be included in this work-up to expedite the diagnosis.

Ultimately, the case of this patient is a cautionary tale of delayed diagnosis of a treatable condition due to inconclusive testing. Earlier diagnosis and initiation of treatment to reduce morbidity may have been achieved with earlier involvement of specialists and a more thoughtful diagnostic testing.
